# Estimating the Excess Mortality Risk during Two Red Alert Periods in Beijing, China

**DOI:** 10.3390/ijerph15010050

**Published:** 2017-12-29

**Authors:** Weilin Zeng, Lingling Lang, Yue Li, Lingchuan Guo, Hualiang Lin, Yonghui Zhang, Tao Liu, Jianpeng Xiao, Xing Li, Yanjun Xu, Xiaojun Xu, Lauren D. Arnold, Erik J. Nelson, Zhengmin Qian, Wenjun Ma

**Affiliations:** 1 Guangdong Provincial Institute of Public Health, Guangdong Provincial Center for Disease Control and Prevention, Guangzhou 511430, China; letitiazeng@foxmail.com (W.Z.); langll1982@163.com (L.L.); glcbzbs@126.com (L.G.); gztt_2002@163.com (T.L.); jpengx@163.com (J.X.); lixing.echo@foxmail.com (X.L.); 2 Jiangxi Medical School of Nanchang University, No. 461, Nanchang 330006, China; liyue53@126.com; 3 Department of Medical Statistics and Epidemiology, School of Public Health, Sun Yat-sen University, Guangzhou 510080, China; linhualiang2002@163.com; 4 Guangdong Provincial Center for Disease Control and Prevention, Guangzhou 511430, China; zyh@cdcp.org.cn; 5 Institute of Chronic Non-Communicable Disease Control and Prevention, Guangdong Provincial Center for Disease Control and Prevention, Guangzhou 511430, China; gdxyj05@21cn.com (Y.X.); xu-yd@163.com (X.X.); 6 College for Public Health and Social Justice, Department of Epidemiology & Biostastics, Saint Louis University, Salus Center/Room 473, 3545 Lafayette Avenue, Saint Louis, MO 63104, USA; lauren.arnold@slu.edu (L.D.A.); zqian2@slu.edu (Z.Q.); 7 Department of Epidemiology and Biostatistics, Indiana University School of Public Health-Bloomington, Bloomington, IN 47405, USA; nelsonej@slu.edu

**Keywords:** particulate matter, air pollution, red alert, smog, mortality, Beijing

## Abstract

The magnitude of excess mortality risk due to exposures to heavy air pollution during the red alert periods in Beijing remains unknown. A health impact assessment tool combined with the PM_2.5_-mortality relationship was applied to estimate the number of excess deaths due to high air pollution exposure during two red alert periods in Beijing, China in December 2015. Daily PM_2.5_ concentration increased from 80.2 µg/m^3^ to 159.8 µg/m^3^ during the first red alert period and from 61.9 µg/m^3^ to 226 µg/m^3^ during the second period in 2015 when compared to daily PM_2.5_ concentrations during the same calendar date of 2013 and 2014. It was estimated that 26 to 42 excessive deaths (including 14 to 34 cardiovascular deaths, and four to 16 respiratory deaths) occurred during the first period, and 40 to 65 excessive deaths (22 to 53 cardiovascular deaths, and six to 13 respiratory deaths) occurred during the second period. The results show that heavy smog may have substantially increased the mortality risk in Beijing, suggesting more stringent air pollution controlling measures should be implemented to protect the public health.

## 1. Introduction

Air pollution is an important public health issue in China. It has been estimated that air pollution has resulted in more than 1.6 million deaths in China each year (4400 each day), accounting for 17% of the nation’s annual deaths [1]. This places air pollution as the third leading cause of death only after cancer and heart disease [1].

Air pollution in Beijing has gained much attention in recent years due to increased energy consumption, emissions, construction activities and urbanization [2]. Blue sky has been seldomly seen in this city [3]. Episodes of intense atmospheric haze have been frequently reported in recent years [3,4]. High levels of air pollution, especially fine particles <2.5 μm in diameter (PM_2.5_), have been responsible for the decreasing air quality and visibility [5,6], and have been associated with solar ultraviolet radiation reaching the earth’s surface [7,8], the nutrient balance and acidity of the soil [9,10] and global climate change [11].

According to the technical regulation on ambient air quality index (AQI), the AQI level is based on six atmospheric pollutants, including sulfur dioxide, nitrogen dioxide, carbon monoxide, ozone, PM_10_ and PM_2.5_. The AQI is divided into six levels, namely, 0–50, 51–100, 101–150, 151–200, 201–300, and greater than 300. When the AQI value is less than 100, the air is deemed to have no effect on daily life, but when the AQI is larger than 200 (equivalent to a PM_2.5_ concentration of larger than 150 μg/m^3^), it can result in heavy adverse health effects. In 2013, the environmental authorities in Beijing instituted a four-tier warning system to reduce emissions during episodes of intense smog [12]. When the air quality index is expected to be ≥200 for at least 72 h, a red alert, the highest pollution level, will be issued [12]. At this highest warning level, road traffic is restricted with alternate-day driving laws (based on license plate), factory work is reduced or halted, and schools are closed. On 7 December 2015, the Beijing government issued the first red alert for a five-day period during 8–12 December [13]. A second red alert warning was issued only one week later, 19–22 December 2015, due to excessive levels of smog [14].

Although the association between PM_2.5_ and mortality has been demonstrated in a great number of studies [15,16], the extent of the acute adverse health effects of air pollution during these intense smog periods remained largely unknown. As such, we applied a health impact assessment based on the PM_2.5_-mortality relationship to estimate the excess deaths associated with the heavy smog episodes in Beijing [17,18].

## 2. Materials and Methods

### 2.1. Study Setting and Air Pollution Data

Beijing is the capital of China with a population of 21.7 million in 2015 [19]. It has a temperate climate with four distinct seasons. The summer is hot and humid, and the winter is cold and dry. The city is divided into 16 districts, which comprise rural, suburban, and urban locales. Six urban districts (Dongcheng, Xicheng, Haidian, Chaoyang, Fengtai and Shijingshan) were selected for this study, as they had higher air pollution levels than other districts and were more directly related to these two red alert episodes [14]. For example, during the first red alert period, the PM_2.5_ concentration was 111.31 μg/m^3^ in the six urban districts and 85.57 μg/m^3^ in the other districts of Beijing; and during the second period, the PM_2.5_ concentration was 120.81 μg/m^3^ in the six urban districts and 83.07 μg/m^3^ in the other districts of Beijing. According to the Beijing statistical yearbook data, the six urban districts of Beijing covered 59.1% population of the whole of Beijing City [19].

Two red alert episodes were observed in this city on 8–12 December 2015 and 19–22 December 2015. We obtained the daily concentrations of ambient PM_2.5_ during the red alert periods and two different reference periods. The first reference (I) was the same calendar dates of 2013 and 2014. The second reference (II) was the maximum daily PM_2.5_ concentration of 75 μg/m^3^ regulated by the Chinese Ambient Air quality Standard [20].

### 2.2. Estimating Mortality Effects

A health impact assessment was conducted to estimate the excess mortality during the two periods using the method proposed by the World Health Organization (WHO) [21]. Specifically, we used population of the six urban districts of Beijing city, baseline mortality, PM_2.5_ concentration during the red alert periods and during the reference periods in the six urban districts, and an exposure-response function. The WHO has reported that the PM_2.5_-mortality relationship is approximately linear [22], our analysis was thus based on the linear relationship between PM_2.5_ and mortality, which was retrieved from the existing short-term PM_2.5_-mortality association studies among the population of Beijing, China [23,24]. For example, in one study, each 10 μg/m^3^ increase in daily PM_2.5_ concentration in Beijing was associated with a 0.28% increase in all-cause mortality, a 0.32% increase in cardiovascular mortality, and a 0.31% increase in respiratory mortality [25]. In another study, Guo et al. [26] found that the relative risk for each 94 μg/m^3^ PM_2.5_ concentration increase was 2.5% (95% CI: 0.6–4.5%) in Beijing. Hence, we calculated a number of estimates of the death counts based on the PM_2.5_-mortality associations obtained from previous studies, which have been employed in one recent study [27].

### 2.3. Differences in PM_2.5_ Concentrations

**Figure 1:**
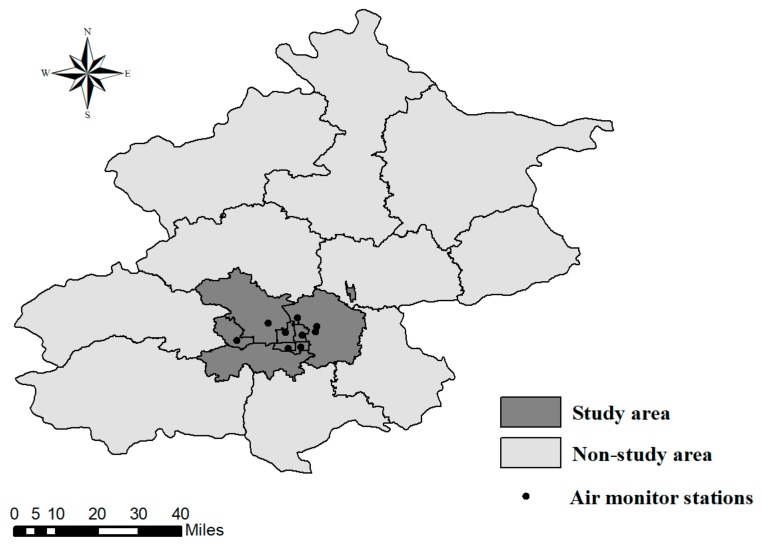
The location of air monitoring stations in the six urban districts of Beijing.

The differences in the PM_2.5_ concentrations during the red alert periods and two different reference periods were compared. PM_2.5_ concentrations levels were collected from the air monitoring stations in Beijing (their locations were shown in Figure 1). The air monitoring data from nine fixed air monitoring stations in the six urban districts were used in this study. These stations were believed to be representative of the air pollution situation in the urban areas of Beijing, and have been used in one previous study [27]. These daily air pollution data were publicly accessible (http://www.cnemc.cn/). Besides PM_2.5_, other air pollutants, such as sulphur dioxide (SO_2_) and nitrogen dioxide (NO_2_) and weather factors were also closely related to mortality in previous studies [23,28]. To clarify the effects of ambient PM_2.5_, we also collected the data of these variables and examined whether there were any differences between the red alert episodes and reference periods.

### 2.4. Baseline Number of Mortality

Based on a population of 12.82 million residents [19] and an overall annual mortality of seven per 1000 in the six urban districts of Beijing in 2015, there was a daily average of 246 overall mortalities. About 43.74% and 12.32% of the overall mortalities were cardiovascular and respiratory diseases in Beijing [29], thus, there were 108 cardiovascular and 30 respiratory deaths each day in the six urban districts of Beijing city. This study was approved by the Institutional Review Board of the Guangdong Provincial Center for Disease Control and Prevention. Informed consent was not required because we did not use any original health information for this analysis.

### 2.5. Statistical Analysis

The relative risk and excessive risk of the associations were firstly transformed into the corresponding regression coefficient (β) for each one μg/m^3^ increase in PM_2.5_ using the following Equation (1):

β = ln(RR)/unit = ln(1 + ER)/unit;(1)

Then the 95% CI for β (β_lower_ and β_upper_) were calculated using Equations (2) and (3):

β_lower_ = ln(RR_lower_)/unit = ln(1 + ER_lower_)/unit, (2)

β_upper_ = ln(RR_upper_)/unit = ln(1 + ER_upper_)/unit. (3)

We then estimated the excess deaths due to the high PM_2.5_ concentrations during the red alert periods using the following Equation (4) [30]:

Δmortality = baseline mortality * [exp(β * ΔPC) − 1](4)

where Δmortality refers to the estimated excessive deaths, and ΔPC is the differences in ambient PM_2.5_ concentrations.

## 3. Results

A comparison of PM_2.5_ concentration during the red alert and two different reference periods in Beijing is summarized in Table 1. During the two red alert periods, Beijing experienced some of the worst air quality on record. The mean PM_2.5_ concentration during the first red alert period was 159.75 μg/m^3^, while the second red alert period had a mean PM_2.5_ concentration of 226.04 μg/m^3^. These PM_2.5_ concentration levels were six and eight times greater, respectively, than the WHO guideline of 25 μg/m^3^ for safe 24-h PM_2.5_ concentration level. During the first red alert period, PM_2.5_ concentration levels were 1.99 times greater (159.75 vs. 99.10 μg/m^3^) compared to the Reference I, and 2.13 times greater (159.75 vs. 75.00 μg/m^3^) when compared to the national PM_2.5_ concentration standard (Reference II). During the second red alert period, PM_2.5_ concentrations were 3.65 times greater (226.04 vs. 61.92 μg/m^3^) compared with Reference I, and 3.01 times greater (226.04 vs. 75.00 μg/m^3^) when compared with Reference II. There was not a significant increase in the concentrations of SO_2_ during the two red alert periods; however, NO_2_ concentrations increased significantly.

Table 2 illustrates the excess mortality as a result of exposure to the high PM_2.5_ concentration during the two smog episodes in Beijing. We found that, during the first red alert period (8–12 December 2015), the excessive all-cause mortality ranged from 26 to 42 deaths using the same date of prior two years as the reference period (Reference I), and 25 to 39 deaths according to Reference II. The estimated number of excess cardiovascular deaths ranged from 14 to 34 using Reference I and from 13 to 32 using Reference II. The estimated excessive respiratory mortality ranged from 4 to 16 using Reference I, and from 4 to 15 using Reference II.

Excess deaths were also estimated during the second red alert period (19–22 December 2015) in Beijing. We found that, during the 4-day heavy pollution period, the increase in all-cause mortality ranged from 40 to 65 deaths based on Reference I, and 35 to 55 deaths based on Reference II. The increase in cardiovascular deaths was estimated to range from 22 to 53 (Reference I), and from 19 to 45 (Reference II); the estimated increase in respiratory deaths ranged from 6 to 13 (Reference I), and from 5 to 11 (Reference II).

For the sensitivity analysis (Table S1), all analyses were repeated using the data of the whole city instead of the six urban districts of Beijing. The results were similar. During the first red alert period, the excessive all-cause mortality ranged from 45 to 71 deaths using Reference I, and 42 to 67 deaths according to Reference II. The estimated number of excess cardiovascular deaths ranged from 24 to 58 using Reference I and from 22 to 54 using Reference II. The estimated excessive respiratory mortality ranged from 6 to 27 using Reference I, and from 6 to 26 using Reference II. During the the second red alert period, the increase in all-cause mortality ranged from 68 to 109 deaths based on Reference I, and 59 to 94 deaths based on Reference II. The increase in cardiovascular deaths was estimated to range from 36 to 89 (Reference I), and from 31 to 76 (Reference II); the estimated increase in respiratory deaths ranged from 10 to 42 (Reference I), and from 8 to 36 (Reference II).

## 4. Discussion

The analyses presented here indicated the magnitude of the mortality effects linked with Beijing’s lethal smog in December 2015, which resulted in the first two occasions of the red alert warning system that was instituted in 2013. This suggests that substantial excess mortality risk was associated with the high levels of particulate matter pollution during the red alert periods in Beijing. Our analyses estimated that about 26–42 and 40–65 excessive mortalities could be attributable to the high PM_2.5_ exposures during the two smog episodes, corresponding to about 2.1–3.4% and 4.1–6.6% of the daily mortalities in the study area.

China’s rapid economic expansion over the last 30 years has been accompanied by increasing air pollution concerns and adverse health effects, which has been a focus of international attention in recent years [33,34,35]. Accumulating evidence has been available on the effects of particulate matter pollution on mortality in the past years [36,37]. Our study estimated the number of deaths associated with the extreme levels of particulate air pollution during the heavy smog episodes in December 2015. The findings are consistent with previous studies that estimated mortality risk during periods of excessive air pollution. For example, it was estimated that about 12,000 excess deaths occurred in London, England between December 1952 and February 1953 due to the acute and prolonged effects of elevated smog levels [38]. Similarly, heavy smog in northern China during January 2013 also resulted in excessive mortality and morbidity [4]. Several mechanisms have been proposed to explain the acute mortality effects of ambient PM_2.5_ exposure. PM_2.5_ have large surface areas and can carry lots of toxic stuffs, entering the respiratory tract with airflow, accumulating there by diffusion, damaging other parts of the body through air exchange in the lungs [39]. This was illustrated by a study which examined the air quality improvement measures during the Beijing Olympics. Rich et al. reported that the improved air quality was responsible for the significant changes in the biomarkers related to the systemic inflammation [40]. Some of the fine particles even had the potential to cross the epithelium and enter the systemic circulation, inducing inflammation at both epithelial and interstitial sites, as well as reach other target sites [41,42]. Individuals with existing cardiovascular and respiratory conditions may be more vulnerable to these effects [43,44].

There were several limitations of this study. First, we estimated only short-term mortality effects due to exposure to high levels of ambient PM_2.5_ during the red alert periods. Long-term mortality effects of PM_2.5_, however could not be estimated due to a lack of the necessary data. Thus, the reported results might have resulted in under-estimation of the mortality effects of the PM_2.5_. Secondly, we used mortality as the health outcome for this analysis, which might have underestimated the health impacts of high PM_2.5_ concentrations during the heavy smog episodes in Beijing, because the health effects of air pollution exposure were related to several medical conditions that were not evaluated in our analysis, such as hospital admissions, emergency department visits, and symptom aggravation, among others [38]. Third, we obtained the estimated effect magnitudes from multiple studies. There studies employed different modelling methods to examine the association between PM_2.5_ and mortality [2,25,26,31,32], making it difficult to compare between studies [45]. It should be noted that we obtained mortality risk estimates for PM_2.5_ from multiple studies conducted in Beijing to account for the variability in estimates. These effect estimates were based on daily routine activity patterns of Beijing residents; however, the residents were advised to avoid normal outdoor activities during the red alert periods; so the actual exposure level of the population might be lower than the outdoor monitoring concentrations, it was thus possible that our analysis had over-estimated the adverse health effects.

## 5. Conclusions

In summary, the results from this study suggests that substantial mortality risk is associated with excessive PM_2.5_ concentration levels during the red alert episodes in Beijing. These findings support that policies and strategies should be adopted to reduce air pollution in Beijing to prevent mortality and morbidity.

## Figures and Tables

**Table 1:** Comparison of the mean PM_2.5_ concentrations between the red alert and reference periods in the six urban districts of Beijing, China.

Air Pollutants	Red Alert Period	Reference I		Reference II	
		Concentration	Change (%)	Concentration	Change (%)
8–12 December 2015					
PM_2.5_ (μg/m^3^)	159.75	80.24	99.10	75.00	113.00
SO_2_ (μg/m^3^)	23.96	29.33	−18.32	150.00	−84.03
NO_2_ (μg/m^3^)	81.76	56.66	44.29	80.00	2.20
19–22 December 2015					
PM_2.5_ (μg/m^3^)	226.04	61.92	265.06	75.00	201.39
SO_2_ (μg/m^3^)	31.88	34.72	−8.16	150.00	−78.75
NO_2_ (μg/m^3^)	104.65	62.28	68.03	80.00	30.81

**Table 2:** Estimated excess mortality due to the severe smog events declared as the red-alert periods (8–12 and 19–22 December 2015) in Beijing.

Type, Reference	Reference I		Reference II	
	ΔMortality	95% CI	ΔMortality	95% CI
8–12 December 2015				
All-cause mortality				
Guo, 2013 [26]	26	6–47	25	6–44
Li, 2014 [2]	42	30–55	39	28–51
Li, 2015 [25]	28	18–41	26	17–38
CVD mortality				
Dong, 2013 [31]	34	3–65	32	3–61
Li, 2015 [25]	14	7–21	13	7–19
Respiratory mortality				
Li, 2013 [32]	16	12–20	15	12–18
Li, 2015 [25]	4	0–8	4	0–7
19–22 December 2015				
All-cause mortality				
Guo, 2013 [26]	40	10–72	35	8–62
Li, 2014 [2]	65	46–84	55	39–72
Li, 2015 [25]	43	28–63	37	24–54
CVD mortality				
Dong, 2013 [31]	53	5–100	45	4–86
Li, 2015 [25]	22	11–32	19	9–27
Respiratory mortality				
Li, 2013 [32]	13	10–16	11	9–14
Li, 2015 [25]	6	0–12	5	0–10

## Supplementary Materials

The following are available online at www.mdpi.com/1660-4601/15/1/50/s1, Table S1: Sensitivity analysis on excess mortality due to the severe smog events declared as the red-alert periods (8–12 and 19–22 December 2015) in the whole Beijing city.
