# Supplementary material for: Estimating the Excess Mortality Risk during Two Red Alert Periods in Beijing, China
Source: Int J Environ Res Public Health. 2017 Dec 29;15(1):50. doi: 10.3390/ijerph15010050 (PMC5800149; doi:10.3390/ijerph15010050)
Supplement: Supplementary file 1 [file ijerph-15-00050-s001.pdf]

Table S1. Sensitivity analysis on excess mortality due to the severe smog events declared as the red-alert periods (8–12 and 19–22 December 2015) in the whole Beijing city.

	Reference I		Reference II	
	**Δ Mortality**	**95% CI**	**Δ Mortality**	**95% CI**
December 8-12, 2015				
**All-cause mortality**				
Guo, 2013	45	11-80	42	10-74
Li, 2014	71	50-93	67	47-87
Li, 2015	48	31-70	44	29-65
**CVD mortality**				
Dong, 2013	58	5-110	54	5-103
Li, 2015	24	12-35	22	11-33
**RESP mortality**				
Li, 2013	27	21-33	26	20-31
Li, 2015	6	0-13	6	0-12
December 19-22, 2015				
**All-cause mortality**				
Guo, 2013	68	17-122	59	14-105
Li, 2014	109	77-143	94	66-122
Li, 2015	73	47-107	62	40-91
**CVD mortality**				
Dong, 2013	89	8-169	76	7-144
Li, 2015	36	18-53	31	16-46
**Respiratory mortality**				
Li, 2013	42	32-51	36	28-44
Li, 2015	10	0-20	8	0-17
